# Coding-complete genome sequence of transmissible gastroenteritis virus isolated from a diarrheic piglet in South Korea

**DOI:** 10.1128/mra.00028-26

**Published:** 2026-06-29

**Authors:** Young-Hyeon Lee, Hyeob Jin, Bo-Kyoung Jung, Song-Yi Kim, Min-Kyung Jang, Gyu-Nam Park, Yun Sang Cho, Dong-Jun An

**Affiliations:** 1 Virus Disease Division, Animal and Plant Quarantine Agency 65359, Gimcheon, Gyeongbuk-do, Republic of Korea; Katholieke Universiteit Leuven, Leuven, Belgium

**Keywords:** TGEV, complete genome, phylogenetic analysis, mutation, deletion

## Abstract

We report the coding-complete genome sequences of a transmissible gastroenteritis virus strain, SOA, isolated from a diarrheic piglet in South Korea, and its serially passaged derivative, SOAp90. Both strains were closely related to the Vietnamese VET-16 strain, and SOAp90 contained multiple mutations.

## ANNOUNCEMENT

Transmissible gastroenteritis virus (TGEV), an *Alphacoronavirus suis* within the family *Coronaviridae,* possesses a positive-sense single-stranded RNA genome of approximately 28.5 kb (1). The genome comprises nine open reading frames (ORFs) organized as 5′UTR–ORF1a–ORF1b–S–ORF3a–ORF3b–E–M–N–ORF7–3′UTR. Although TGEV outbreaks have become infrequent in South Korea, genetically diverse variants continue to emerge in Europe and China due to rapid evolution and recombination (2, 3). Notably, TGEV is known to undergo gene deletions and mutations during serial passage in hosts or cell cultures, which may alter viral pathogenicity (4–6). Here, we report the coding-complete genome sequences of a TGEV strain, SOA, isolated in South Korea, and its 90th-passage derivative, SOAp90.

The SOA strain was isolated using swine testicular (ST) cells inoculated with a fecal sample collected from a pig farm in Gyeongbuk Province, South Korea, where TGEV-positive diarrhea was confirmed in 2017. ST cells were maintained at 37°C with 5% CO₂ in Dulbecco’s modified Eagle medium (Gibco, USA) supplemented with 10% fetal bovine serum (FBS) and 1% antibiotic–antimycotic solution (Gibco, USA). Viral RNA was extracted using the RNeasy Mini Kit (Qiagen, Germany). The coding-complete genome was amplified by one-step reverse transcription-PCR(RT-PCR) using the HelixCript One-Step RT-PCR Kit (NanoHelix, Korea) with TGEV-specific universal primers (7), generating 44 overlapping amplicons. PCR products were purified using the Wizard SV Gel and PCR Clean-Up System (Promega, USA) and subjected to Sanger sequencing. No next-generation sequencing platform was used. Sequences were assembled using BioEdit (v7.2.5), and the nine ORFs were identified and annotated by comparison with reference TGEV genomes in GenBank.

**Fig 1:**
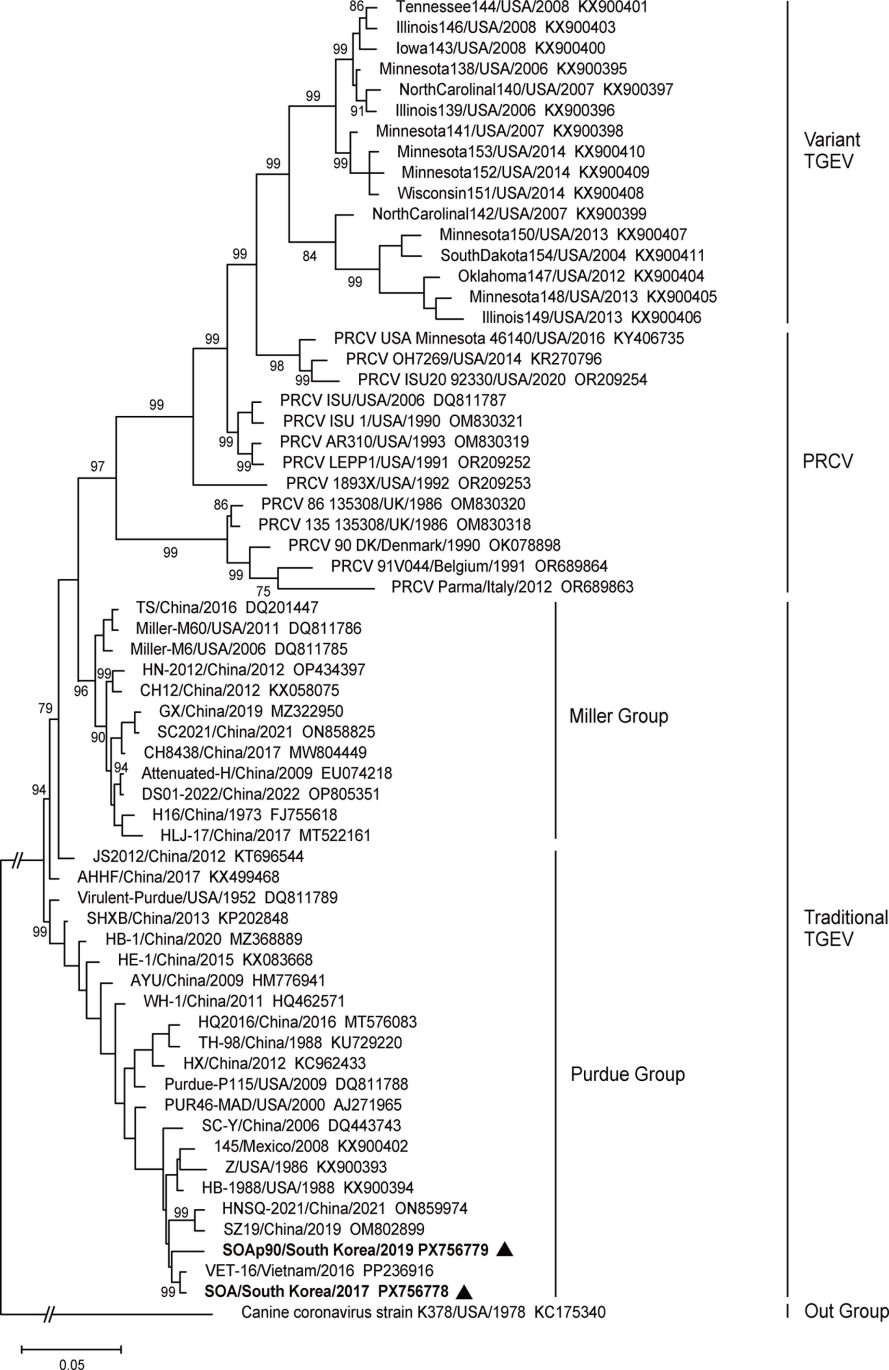
Phylogenetic analysis based on the spike gene from SOA, SOAp90, and 62 reference strains. Multiple sequence alignments were generated using ClustalW in BioEdit with default parameters, and the phylogenetic tree was constructed using the maximum-likelihood method (based on the Tamura-Nei model) in MEGA X (v10.2.6) and tested using 1,000 bootstrap values. The two Korean strains isolated in this study are denoted by black triangles. Canine coronavirus K387 strain was used as an outgroup.

The coding-complete genome sequences of strains SOA and SOAp90 were determined to be 28,596 nucleotides (nt) (GC content, 37.5%) and 27,956 nt (GC content, 37.7%) in length, respectively, sharing 99.89% nucleotide identity across the aligned overlapping regions, excluding gaps. Genome-wide comparison showed that both strains were most closely related to the Chinese TGEV strain WH-1 (GenBank accession number HQ462571.1), with nucleotide identities of 99.84% for SOA and 99.82% for SOAp90. The spike (*S*) gene, a major determinant of host interaction, showed 99.61% nucleotide identity between SOA and SOAp90. Interestingly, the *S* genes of both strains exhibited the highest sequence similarity to the Vietnamese strain VET-16 (SOA, 99.91%; SOAp90, 99.70%; GenBank accession number PP236916.1), rather than to WH-1. Phylogenetic analysis placed the Korean isolate within the TGEV Purdue subtype, clustering closely with the VET-16 strain (Fig. 1). These results indicate that SOA and SOAp90 have potential *S* gene recombination.

Comparative genomic analysis identified 17 nucleotide substitutions in the *S* gene of SOAp90 relative to the parental SOA strain, resulting in amino acid changes, including N380E, Y402H, H438Y, and S673H, within antigenic site D. In addition, a 632-nt deletion was detected in the ORF3 region of SOAp90. This deletion pattern is similar to that observed in the VET-16 strain and distinguishes SOAp90 from classical TGEV strains, suggesting genomic alterations associated with serial passage.

## Data Availability

The coding-complete genome sequences of TGEV strains SOA and SOAp90 have been deposited in GenBank under accession numbers PX756778 for SOA and PX756779 for SOAp90. The Sanger sequencing data have been deposited in the Sequence Read Archive under BioProject PRJNA1468846, and BioSample accession numbers SAMN60306055 for SOA and SAMN60306056 for SOAp90.
